# Emergency Department Visits and Hospitalizations for Selected Nonfatal Injuries Among Adults Aged ≥65 Years — United States, 2018

**DOI:** 10.15585/mmwr.mm7018a1

**Published:** 2021-05-07

**Authors:** Briana Moreland, Robin Lee

**Affiliations:** ^1^Synergy America, Inc., Duluth, Georgia; ^2^Division of Injury Prevention, National Center for Injury Prevention and Control, CDC.

Approximately 60,000 older adults (aged ≥65 years) die from unintentional injuries each year; in 2019 these included 34,000 fall deaths, 8,000 traffic-related motor vehicle crash deaths, and 3,000 drug poisoning deaths (*1*). In addition, >9,000 suicide deaths occur among older adults each year (*1*). Deaths among older adults account for 33% of these unintentional injury deaths and 19% of suicide deaths among all age groups (*1*). Nonfatal injuries from these causes are more common in this age group and can lead to long-term health consequences, such as brain injury and loss of independence. This study included 2018 data from the Agency for Healthcare Research and Quality’s Healthcare Cost and Utilization Project (HCUP) to determine the prevalence of selected nonfatal injuries among older adults treated in emergency departments (EDs) and hospitals. Injury mechanisms among the leading causes of injury death in older adults were studied, including unintentional falls, unintentional traffic-related motor vehicle crashes, unintentional opioid overdoses, and self-harm (suicidal and nonsuicidal by any mechanism). In 2018, an estimated 2.4 million ED visits and >700,000 hospitalizations from these injuries occurred among adults aged ≥65 years. Unintentional falls accounted for >90% of the selected ED visits and hospitalizations. Injuries among older adults can be prevented (*2*). Educational campaigns, such as CDC’s Still Going Strong* awareness campaign, that use positive messages can encourage older adults to take steps to prevent injuries. Health care providers can help prevent injuries by recommending that older patients participate in effective interventions, including referrals to physical therapy and deprescribing certain medications.^†^

Data from the 2018 HCUP Nationwide Emergency Department Sample (NEDS) and National Inpatient Sample (NIS) were analyzed for rates of nonfatal injuries resulting in ED visits and hospitalizations among adults aged ≥65 years.^§^ NEDS included data from 990 hospital EDs across 36 U.S. states and the District of Columbia. NIS included data from 47 participating states and the District of Columbia, which covered >97% of the U.S. population. ED visit diagnosis codes selected for analysis were *International Classification of Diseases, Tenth Revision, Clinical Modification* (ICD-10-CM) injury codes in any position^¶^ and an ICD-10-CM code of one of the following injury mechanisms: unintentional falls,** unintentional traffic-related motor vehicle crashes,^††^ or self-harm^§§^ in any position. Hospitalizations were selected if the primary diagnosis was an injury (ICD-10-CM injury code) and one of the aforementioned injury mechanisms (ICD-10-CM code in any position).^¶¶^ ED visits and hospitalizations were considered unintentional opioid overdose–related if ICD-10-CM codes or ICD-10 Procedure Coding System codes (used to collect inpatient procedures) for unintentional opioid overdoses*** were present in any field (*3*). To identify the full effect of selected injuries on ED visits and hospitals, all encounter types (initial, subsequent, and sequalae) were included. Among the selected injuries, 98.4% of ED visits and 94.1% of hospitalizations were for an initial encounter. Overdose visits were limited to opioid overdose (prescription and heroin) because opioid use is related to the other injuries included in this study and opioids are frequently prescribed to older adults (*3*,*4*). ED visits and hospitalizations with missing sex or age data or those resulting in deaths were excluded, as were ED visits leading to patient hospitalizations to avoid overlap between data systems. This activity was reviewed by CDC and was conducted consistent with applicable federal law and CDC policy.^†††^

ED visits and hospitalizations were weighted to represent the U.S. population using survey procedures in SAS statistical software (version 9.4; SAS Institute). Rates of ED visits and hospitalizations were age-adjusted to the 2000 U.S. standard population using the direct method. Injuries were analyzed by sex and age group (65–74, 75–84, and ≥85 years). T-tests were used for selected pairwise comparisons; p-values <0.05 were considered statistically significant.

In 2018, an estimated 2.4 million ED visits among adults aged ≥65 years (4,744 per 100,000) were associated with unintentional falls, unintentional motor vehicle crashes, unintentional opioid overdoses, and self-harm (Table). Unintentional falls accounted for 91.8% of the selected injury-related ED visits, followed by unintentional crashes (7.8%). The rate of ED visits for unintentional fall injuries among women (5,003 per 100,000) was 41.7% higher than that among men (3,530 per 100,000). Rates of ED visits for unintentional fall injuries per 100,000 persons increased with age, from 2,678 among adults aged 65–75 years to 4,900 among adults aged 75–84 years and 9,867 among adults aged ≥85 years. Unintentional crash injury–related ED visits decreased with age; among adults aged 65–74, 75–84, and ≥85 years, ED visits per 100,000 persons were 401, 337, and 236, respectively. The rate of unintentional opioid overdose–related ED visits was higher among men (18 per 100,000) than among women (14 per 100,000). Compared with other older adults, those aged 65–74 years had the highest rates of visits for unintentional opioid overdose (20 per 100,000) and self-harm (13 per 100,000).

**TABLE:** Rates* of injury-related emergency department visits^†^ and hospitalizations**^§^** for selected causes^¶^ among adults aged ≥65 years, by cause, sex, and age group — Healthcare Cost and Utilization Project, Nationwide Emergency Department Sample and National Inpatient Sample, United States, 2018

Cause, sex, and age group	ED visits			Hospitalizations		
	Weighted no.	Rate	(95% CI)	Weighted no.	Rate	(95% CI)
All causes**						
**Total^††^**	**2,419,788**	**4,744.0**	**(4,623.1–4,864.8)**	**717,660**	**1,425.3**	**(1,404.2–1,446.3)**
**Sex**						
Male (ref)	844,954	3,904.1	(3,799.5–4,008.8)	244,890	1,160.5	(1,140.3–1,180.7)
Female	1,574,834	5,393.3^§§^	(5,257.1–5,529.6)	472,770	1,609.6^§§^	(1,585.5–1,633.7)
**Age group, yrs**						
65–74 (ref)	948,492	3,110.6	(2,951.3–3,269.9)	206,755	678.1	(657.3–698.8)
75–84	808,813	5,253.9^§§^	(4,966.0–5,541.9)	250,925	1,630.0^§§^	(1,582.6–1,677.4)
≥85	662,483	10,122.7^§§^	(9,505.9–10,739.6)	259,980	3,972.5^§§^	(3,851.4–4,093.6)
**Unintentional falls^¶¶^**						
Total^††^	2,216,681	4,362.1	(4,249.3–4,474.9)	654,895	1,305.2	(1,285.6–1,324.8)
**Sex**						
Male (ref)	755,836	3,529.8	(3,432.6–3,626.9)	215,540	1,034.6	(1,016.2–1,052.9)
Female	1,460,845	5,002.6^§§^	(4,875.0–5,130.2)	439,355	1,494.0^§§^	(1,471.3–1,516.8)
**Age group, yrs**						
65–74 (ref)	816,650	2,678.2	(2,541.9–2,814.6)	170,985	560.7	(543.8–577.7)
75–84	754,281	4,899.7^§§^	(4,630.7–5,168.7)	231,490	1,503.7^§§^	(1,460.6–1,546.9)
≥85	645,750	9,867.1^§§^	(9,265.2–10,468.9)	252,420	3,857.0^§§^	(3,739.6–3,974.4)
Unintentional motor vehicle crashes***						
Total^††^	189,531	357.2	(345.9–368.4)	42,040	81.4	(78.7–84.2)
**Sex**						
Male (ref)	82,493	347.7	(336.5–358.9)	20,880	90.8	(87.1–94.5)
Female	107,038	367.6^§§^	(355.3–379.8)	21,160	74.0^§§^	(71.0–76.9)
**Age group, yrs**						
65–74 (ref)	122,238	400.9	(375.9–425.8)	21,915	71.9	(67.3–76.5)
75–84	51,828	336.7^§§^	(315.6–357.7)	14,295	92.9^§§^	(86.6–99.1)
≥85	15,465	236.3^§§^	(218.6–254.0)	5,830	89.1^§§^	(82.0–96.1)
**Unintentional opioid overdoses^†††^**						
Total^††^	8,767	16.0	(14.9–17.1)	14,440	27.1	(26.0–28.1)
**Sex**						
Male (ref)	4,529	17.9	(16.0–19.8)	5,780	23.9	(22.5–25.4)
Female	4,238	14.2^§§^	(13.2–15.3)	8,660	29.6^§§^	(28.1–31.0)
**Age group, yrs**						
65–74 (ref)	6,204	20.3	(18.0–22.6)	9,510	31.2	(29.5–32.8)
75–84	1,730	11.2^§§^	(10.0–12.5)	3,730	24.2^§§^	(22.3–26.1)
≥85	834	12.7^§§^	(10.6–14.9)	1,200	18.3^§§^	(15.9–20.7)
**Self-harm^§§§^**						
Total^††^	5,782	10.6	(9.9–11.4)	8,420	15.7	(14.9–16.5)
**Sex**						
Male (ref)	2,534	10.7	(9.7–11.6)	3,580	15.1	(14.0–16.2)
Female	3,248	10.8	(9.9–11.7)	4,840	16.4	(15.3–17.4)
**Age group, yrs**						
65–74 (ref)	3,908	12.8	(11.6–14.1)	5,490	18.0	(16.9–19.2)
75–84	1,289	8.4^§§^	(7.2–9.5)	2,065	13.4^§§^	(12.0–14.8)
≥85	585	8.9^§§^	(7.2–10.7)	865	13.2^§§^	(11.2–15.2)

**Abbreviations:** CI = confidence interval, ED = emergency department; ref = reference group.

* ED visits/hospitalizations per 100,000. Rates are age adjusted using the 2000 U.S. standard population except for the age group-specific rates.

^†^ Records for patients who were hospitalized or died in the ED were excluded; weighted number estimates were weighted to be representative of the U.S. population.

^§^ Records were excluded if the patient died in the hospital; weighted number estimates were weighted to be representative of the United States.

^¶^ For all the selected injuries, observations were included for all encounters (initial, subsequent, and sequalae).

** All causes category represents the number of unique injury visits for unintentional falls, unintentional motor vehicle crashes, unintentional opioid overdoses, and self-harm. The sum of the individual mechanisms is higher than the total because some injury-related emergency department visits and hospitalizations had more than one mechanism of injury code.

**^††^** Totals for each mechanism of injury might not sum to totals across sex and age group because of rounding the weighted estimates.

^§§^ P-value <0.05 when compared with reference group by t-test.

^¶¶^ ICD-10-CM codes were the following: injury diagnosis code in any position (ED) or primary position (hospital) M97, S00–S99, T07–T34, T36–T76 (T36–T50 with sixth character = 1–4, except T36.9, T37.9, T39.9, T41.4, T42.7, T43.9, T45.9, T47.9, and T49.9 with a fifth character = 1–4), T79; and an unintentional fall code in any position: V00.11–V00.89 (with sixth character = 1), W00–W17 (W16 with a sixth character = 2, except W16.4 and W16.9 with a fifth character = 2), W18.1–W18.3, and W19.

*** ICD-10-CM codes were the following: injury diagnosis code in any position (ED) or primary position (hospital) M97, S00–S99, T07–T34, T36–T76 (T36–T50 with sixth character = 1–4, except T36.9, T37.9, T39.9, T41.4, T42.7, T43.9, T45.9, T47.9, and T49.9 with a fifth character = 1–4), T79; and an unintentional motor vehicle crash code in any position: V02–V04 (.1 or .9), V09.2, V09.3, V12–V14 (.3–.9), V19.4–V19.6, V19.9, V20–V28 (.3–.9), V29.4–V29.9, V30–V79 (.4–.9), V80.3–V80.5, V81.1, V82.1, V83–V86 (.0–.3), V87.0–V87.8, V89.2 in any position.

^†††^. ICD-10-CM codes were the following: T40.0X1, T40.1X1, T40.2X1, T40.3X1, T40.4X1, T40.601, and T40.691.

^§§§^ ICD-10-CM codes were the following: injury diagnosis code in any position (EDs) or primary position (hospital) M97, S00–S99, T07–T34, T36–T76 (T36–T50 with sixth character = 1–4, except T36.9, T37.9, T39.9, T41.4, T42.7, T43.9, T45.9, T47.9, and T49.9 with a fifth character = 1–4), T79; and a self-harm code in any position: T36–T65 with sixth character = 2 (except for T36.9, T37.9, T39.9, T41.4, T42.7, T43.9, T45.9, T47.9, T49.9, T51.9, T52.9, T53.9, T54.9, T56.9, T57.9, T58.0, T58.1, T58.9, T59.9, T60.9, T61.0, T61.1, T61.9, T62.9, T63.9, T64.0, T64.8, and T65.9 with fifth character = 2), T71 with sixth character = 2, T14.91, and X71–X83.

In 2018, >700,000 hospitalizations associated with unintentional falls, unintentional motor vehicle crashes, unintentional opioid overdoses, and self-harm occurred among older adults. Unintentional falls accounted for 91.3% of the selected injury-related hospitalizations followed by unintentional motor vehicle crashes (5.9%). The rate of hospitalizations for unintentional falls was higher among women (1,494 per 100,000) than among men (1,035 per 100,000), and increased with age; among adults aged 65–74, 75–84, and ≥85 years, hospitalization rates for unintentional fall–related injuries were 561, 1,504, and 3,857 per 100,000, respectively. Hospitalization rate of unintentional motor vehicle crashes was higher among men (91 per 100,000) than among women (74 per 100,000), whereas the rate of unintentional opioid overdose–related hospitalizations was higher among women (30 per 100,000) than among men (24 per 100,000). Unintentional opioid overdose–related hospitalizations decreased with age: among adults aged 65–74, 75–84, and ≥85 years, hospitalization rates per 100,000 were 31, 24, and 18, respectively. Hospitalization rates for unintentional opioid overdose (27 per 100,000) and self-harm (16 per 100,000) were higher than rates of ED visits during which patients were treated and released (16 and 11 per 100,000, respectively).

## Discussion

In 2018, injuries from unintentional falls, unintentional motor vehicle crashes, unintentional opioid overdoses, and self-harm among adults aged ≥65 years were associated with an estimated 2.4 million ED visits and >700,000 hospitalizations. Unintentional falls accounted for >90% of these visits. Women had higher rates of fall-related injury ED visits and hospitalizations than did men. Although women are more likely to report fall injuries, fall-related mortality rates are higher in men than in women (*5*). The relationship between sex and fall-related injuries has not been fully explained. In this study, rates of ED visits and hospitalizations for fall-related injuries increased with age. Many risk factors for injuries increase with age, including poor balance, visual impairment, and increased medication use (*5*).

Motor vehicle crash injuries are related to visual impairment, use of certain medications, and frailty (*6*). This study found that ED visits for crash-related injuries decreased with age, perhaps because fewer older adults drive or ride in a car as they age (*7*). Older men constitute a higher percentage of drivers than do older women (*7*). This might partially explain the higher rates of crash-related hospitalizations among men in the present study.

Although injuries from opioid overdoses or self-harm were less common than were injuries from falls or motor vehicle crashes, these injury mechanisms share common risk factors. Depression has been associated with opioid use, self-harm, and falls among older adults (*3*,*5*,*8*). Opioid use is associated with an increased risk for falls and motor vehicle crashes (*4*). Poisoning, the most common mechanism of self-harm among older adults (*8*), often includes medications linked to falls, including opioids, benzodiazepines, and tricyclic antidepressants (*4*,*5*,*9*). Managing these shared risk factors can help prevent injuries.

The findings in this report are subject to at least seven limitations. First, this study examined a subset of common nonfatal injuries; therefore, not all nonfatal injuries among older adults are represented. Second, injury-related ED visits and hospitalizations could have multiple ICD-10-CM mechanism of injury codes, causing some injuries (<1%) to be attributed to multiple mechanisms. Third, ED visits for falls, crashes, and self-harm were included only if both an injury diagnosis ICD-10-CM code and a mechanism of injury ICD-10-CM code were present, leading to a possible underestimation of injury-related ED visits. Fourth, hospitalizations for falls, motor vehicle crashes, and self-harm were included only if the primary diagnosis was an injury. This could underestimate rates of injury-related hospitalizations. Fifth, injury visits should not be interpreted to represent individual patients because all encounter types were counted, which could include multiple visits for a single injury. Sixth, this analysis was specific to unintentional opioid overdoses, which account for approximately 53% of unintentional overdose deaths among older adults.^§§§^ Finally, injuries of undetermined intent were not included in this analysis, which could lead to an underestimation of injury rates for which intent is difficult to determine, such as opioid overdose.

Injuries are not an inevitable part of aging and can be prevented (*10*). CDC’s Still Going Strong awareness campaign can guide older adults about simple steps to avoid injuries as they age. Important steps include exercises to improve strength and mobility, regular eye exams, and speaking with a health care provider about reducing medications that can increase the risk for injury, such as benzodiazepines, opioids, and antidepressants.^¶¶¶^ CDC also offers tools to help health care providers and their older patients prevent injuries and deaths from falls,**** motor vehicle crashes,^††††^ opioid overdoses,^§§§§^ and suicide.^¶¶¶¶^ Resources such as these can help reduce common injuries among older populations and reduce the number of injuries that require medical treatment.

**Summary**

**What is already known about this topic?**

Injuries are a leading cause of death among U.S. adults aged ≥65 years; nonfatal injuries among this age group are more common and result in long-term health consequences, including brain injuries or the loss of independence.

**What is added by this report?**

In 2018, an estimated 2.4 million emergency department visits and >700,000 hospitalizations occurred among older adults as a result of injuries from falls, motor vehicle crashes, opioid overdoses, and self-harm. Unintentional falls accounted for >90% of these visits.

**What are the implications for public health practice?**

Injuries are not an inevitable part of aging. Educational campaigns that use positive messages can encourage older adults to speak with their health care provider about preventing injuries. Health care providers can help prevent injuries by referring to physical therapy and deprescribing certain medications.

## Footnotes

*
https://www.cdc.gov/StillGoingStrong

†
https://www.cdc.gov/steadi/index.html

§
https://www.hcup-us.ahrq.gov/

¶ ICD-10-CM codes: M97, S00–S99; T07–T34, T36–T76 (T36–T50 with sixth character = 1–4, except T36.9, T37.9, T39.9, T41.4, T42.7, T43.9, T45.9, T47.9, and T49.9, with a fifth character = 1–4), and T79. Codes are seven characters long, with the last character representing the encounter type. HCUP-NEDS records include up to 35 diagnosis codes; HCUP-NIS contains up to 40 diagnosis codes for each visit.

** Unintentional fall ICD-10-CM codes: V00.11–V00.89 (with sixth character = 1), W00–W17 (W16 with sixth character = 2, except W16.4 and W16.9 with fifth character = 2), W18.1–W18.3, and W19.

†† Unintentional motor vehicle-traffic-related crash injury ICD-10-CM codes: V02–V04 (0.1 or 0.9), V09.2, V09.3, V12–V14 (.3–.9), V19.4–V19.6, V19.9, V20–V28 (.3–.9), V29.4–V29.9, V30–V79 (.4–.9), V80.3–V80.5, V81.1, V82.1, V83–V86 (.0–.3), V87.0–V87.8, and V89.2.

§§ Self-harm ICD-10-CM codes: T36–T65 with sixth character = 2 (except for T36.9, T37.9, T39.9, T41.4, T42.7, T43.9, T45.9, T47.9, T49.9, T51.9, T52.9, T53.9, T54.9, T56.9, T57.9, T58.0, T58.1, T58.9, T59.9, T60.9, T61.0, T61.1, T61.9, T62.9, T63.9, T64.0, T64.8, and T65.9 with fifth character = 2), T71 with sixth character = 2, T14.91, and X71–X83.

¶¶
https://resources.cste.org/Injury-Surveillance-Methods-Toolkit/Home/GeneralInjuryIndicators

*** Unintentional opioid overdose ICD-10-CM/ICD-10-PCS codes: T40.0X1, T40.1X1, T40.2X1, T40.3X1, T40.4X1, T40.601, and T40.691.

††† 45 C.F.R. part 46.102(l)(2), 21 C.F.R. part 56; 42 U.S.C. Sect. 241(d); 5 U.S.C. Sect. 552a; 44 U.S.C. Sect. 3501 et seq.

§§§
https://wonder.cdc.gov/

¶¶¶
https://doi.org/10.1111/jgs.15767

****
https://www.cdc.gov/steadi/index.html

††††
https://www.cdc.gov/motorvehiclesafety/pdf/older_adult_drivers/CDC-AdultMobilityTool-9.27.pdf

§§§§
https://www.cdc.gov/drugoverdose/pubs/featured-topics/evidence-based-strategies.html

¶¶¶¶
https://www.cdc.gov/violenceprevention/pdf/suicideTechnicalPackage.pdf
