# *Erratum:* Vol. 70, No. 18

**DOI:** 10.15585/mmwr.mm7028a3

**Published:** 2021-07-16

In the report “Emergency Department Visits and Hospitalizations for Selected Nonfatal Injuries Among Adults Aged ≥65 Years — United States, 2018,” on page 663, in the first paragraph, the third complete sentence should have read, “Rates of ED visits for unintentional fall injuries per 100,000 persons increased with age, from 2,678 among adults aged 65–**74** years to 4,900 among adults aged 75–84 years and 9,867 among adults aged ≥85 years.”

